# Rhabdomyolysis secondary to chlorpheniramine maleate toxicity: a case report

**DOI:** 10.3389/fphar.2026.1770052

**Published:** 2026-02-19

**Authors:** Anyang Lu, Rong Wu

**Affiliations:** Department of Emergency, Mianyang Central Hospital, Mianyang, Sichuan, China

**Keywords:** acute kidney injury, antihistamine, chlorpheniramine maleate, poisoning, rhabdomyolysis

## Abstract

**Background:**

Chlorpheniramine maleate is a commonly used antiallergic drug; excessive ingestion can cause adverse effects such as hallucinations, tachycardia, central nervous system depression, and organ dysfunction. However, rhabdomyolysis induced by this drug is rarely reported. This study presents a case of rhabdomyolysis secondary to chlorpheniramine maleate poisoning.

**Case Report:**

A 14-year-old female student developed palpitations after oral ingestion of 100 tablets of chlorpheniramine maleate (4 mg per tablet). Six hours post-ingestion, laboratory tests showed elevated concentrations of myoglobin (>1,200 ng/mL) and creatine kinase (1529.7 U/L). Nine hours post-ingestion, the myoglobin concentration gradually decreased to 889 ng/mL, while the creatine kinase concentration continued to rise to 7,315 U/L. One day after ingestion, the myoglobin concentration further decreased, whereas the creatine kinase concentration peaked at 11,219 U/L. Indicators including troponin, creatine kinase-MB mass, serum creatinine, and serum uric acid remained within the normal range. Following treatment with gastrointestinal decontamination, diuresis, and maintenance of electrolyte balance, the patient’s condition improved and she was discharged.

**Discussion:**

Chlorpheniramine maleate poisoning can cause hallucinations, hypertension, tachycardia, organ dysfunction, and central nervous system depression. Additionally, it may be a potential etiological factor for rhabdomyolysis.

## 1 Introduction

Chlorpheniramine maleate is a classic H1 receptor antagonist that counteracts allergic reaction-induced capillary dilation, reduces capillary permeability, and relieves wheezing caused by bronchial smooth muscle contraction. It exhibits a relatively long-acting antihistaminic effect and also exerts significant central nervous system (CNS) depressant effects. Notably, it can potentiate the actions of anesthetics, analgesics, hypnotics, and local anesthetics. The liver serves as its primary metabolic organ. As a commonly used over-the-counter (OTC) antihistamine in clinical practice, it is typically indicated for the management of allergic reactions; however, its toxic side effects are often overlooked. In cases of overdose or toxicity, it may lead to CNS depression, organ dysfunction, and other manifestations (Bleehen et al., 1987; Joung et al., 2019). Rhabdomyolysis arises from skeletal muscle cell damage due to various etiologies, wherein muscle cells release creatine kinase (CK) and myoglobin into the bloodstream, resulting in a clinical syndrome characterized primarily by acute kidney injury (AKI),Clinical manifestations were variable, Classic clinical manifestations primarily include the following: myalgia, muscle weakness, urine discoloration, and oliguria or anuria, all precipitated by inciting factors such as trauma, strenuous exercise, medication exposure, or infection, Mild rhabdomyolysis might present with no specific clinical symptoms, whereas severe cases could result in serious complications including acute kidney injury and disseminated intravascular coagulation (Bagley et al., 2007). Its etiology and pathogenesis are complex, and its clinical manifestations are often atypical—frequently leading to misdiagnosis or missed diagnosis (Huerta-Alardín et al., 2004; Cervellin et al., 2010). At the time, no unified diagnostic criteria existed for rhabdomyolysis. Several studies suggested that a serum creatine kinase (CK) level exceeding 1,000 U/L,or at least five times the upper limit of normal, could serve as a diagnostic criterion (Stahl et al., 2020). Creatine kinase is predominantly localized in striated muscle; upon myolysis of striated muscle fibers, the enzyme is released into the systemic circulation in large quantities,and its serum concentration correlates directly with the severity and extent of skeletal muscle cell injury. A large-sample study demonstrated a positive correlation between serum creatine kinase levels and the clinical severity of rhabdomyolysis:higher CK levels were associated with a marked increase in the risk of renal injury and the need for dialysis (*p* < 0.001). When serum creatine kinase levels exceeded 5,000 U/L,the risk of renal injury rose significantly (Zhou et al., 2025; Jehle et al., 2025). Our department recently admitted a patient with chlorpheniramine maleate tablet poisoning following oral ingestion. While the patient’s initial toxic symptoms were relatively mild, rhabdomyolysis developed 6 h post-poisoning. This case is reported as follows.

## 2 Case description

The patient was a 14-year-old female student who ingested 100 tablets of chlorpheniramine maleate (4 mg per tablet, Manufacturer:Xinxiang Changle Pharmaceutical Co., Ltd.) on 21 September 2025, due to family conflicts. Following ingestion,she developed palpitations and generalized malaise without other accompanying symptoms. Approximately 6 h post-ingestion,the patient was found by her family and transported to a local hospital, where she was diagnosed with chlorpheniramine maleate toxicity. Prompt gastric lavage and fluid resuscitation were performed immediately. Laboratory studies at the local hospital revealed: serum myoglobin >1,200 ng/mL and creatine kinase (CK) 1529.7 U/L. She was subsequently transferred to our hospital on the same day for further management.

On admission, vital signs were as follows: temperature 36.4 °C, pulse 105 beats per minute, respiratory rate 18 breaths per minute, blood pressure 143/78 mmHg, random blood glucose 5.2 mmol/L,and oxygen saturation (SpO_2_) 99% on room air. The patient was conscious, cooperative,and in acute distress with a normal mental status and appropriate responsiveness. She had a normal body habitus. Physical examination findings included:bilateral pupils equal and round, 3 mm in diameter,and briskly light-responsive; no bulbar conjunctival edema or cyanosis of the lips; trachea midline; symmetrical thorax without obvious retractions; resonant percussion notes over both lung fields; clear bilateral breath sounds without rales or wheezes. Cardiac auscultation revealed a regular rhythm at 105 beats per minute, with no pathological murmurs detected across all valve areas. The abdomen was soft, non-tender to palpation, with no hepatosplenomegaly (liver and spleen not palpable below the costal margins) and negative shifting dullness. Spinal and extremity movements were intact, with no lower extremity edema. Laboratory Tests: Complete blood count (CBC),liver function tests, renal function tests, electrolytes,coagulation function (negative); creatine kinase (CK): 7,315 U/L; cardiac biomarkers:creatine kinase-MB mass (CK-MBmass): 3.24 μg/L, myoglobin (Mb): 889 μg/L, high-sensitivity troponin T (hsTnT):<3 ng/L. Diagnosis: Moderate oral chlorpheniramine maleate poisoning; rhabdomyolysis. Upon admission, the patient was placed on electrocardiographic (ECG) monitoring and underwent determination of plasma chlorpheniramine concentrations [detection method:liquid chromatography-tandem mass spectrometry (LC-MS/MS)]. Treatment included administration of 50 g of activated charcoal for adsorption, 250 mL of 20% mannitol for catharsis, fluid resuscitation, diuresis,electrolyte balance was maintained and symptomatic management. Laboratory testing was repeated on hospital days 2, 3, and 5. Plasma chlorpheniramine assay results were reported on hospital day 5, with a measured concentration of 379.75 ng/mL. Following treatment, the patient was asymptomatic on hospital day 5 and was subsequently scheduled for outpatient follow-up. Laboratory test results at various time points post-poisoning are presented in Table 1.

**TABLE 1:** Laboratory test results at various time points after poisoning.

Test indicators	Inspection time				
	Day 1 6 h post-poisoning	Day 1 9 h post-poisoning	Day 2	Day 3	Day 5
Complete blood count	Normal range	Normal range	Normal range	Normal range	-
Urinalysis	Normal range	Normal range	Normal range	Normal range	-
Coagulation studies	Normal range	Normal range	Normal range	Normal range	-
Alanine transaminase (ALT)U/L Local hospital:6–29U/L Our hospital: 6–29U/L	8	11	14	12	-
Aspartate transaminase (AST)U/L Local hospital:10∼31U/L Our hospital:10∼31U/L	18	30	42	29	-
Total bilirubin (TBIL) µmol/L Local hospital:0∼21 µmol/L Our hospital:0∼21 µmol/L	9.8	9.9	17.7	13.8	-
Serum urea (Urea)mmol/L Local hospital:2.8∼8.2 mmol/L Our hospital:2.5∼6.5 mmol/L	2.87	2.31	-	1.49	-
Serum creatinine (Cr)µmol/L Local hospital:44∼106 µmol/L Our hospital:33∼75 µmol/L	42.8	50.3	-	53.4	-
Serum uric acid (UA)µmol/L Local hospital:150∼360 µmol/L; Our hospital:142.8∼339.2 µmol/L	281.6	231.6	-	222.8	-
Serum potassium (K^+)^mmol/L Local hospital:3.5–5.3 mmol/L Our hospital:3.7–5.2 mmol/L	3.21	3.73	3.08	3.17	-
Creatine kinase (CK)U/L Local hospital:40–200U/L Our hospital:40–200U/L	1,529.7	7,315	11,219	6,149	1,469
Creatine kinase-MB mass (CK-Mbmass)µg/L Local hospital:0–3.1 µg/L Our hospital:0–4.88 µg/L	2.14	3.24	2.59	1.25	-
Myoglobin (Mb)µg/L Local hospital:<59.5 µg/L Our hospital:25–58 µg/L	>1,200	889	73.4	34.3	-
High-sensitivity troponin T (hsTnT)ng/L Local hospital:<0.03 ng/L Our hospital:0–14 ng/L	<0.01	<3	<3	<3	-

“-” indicates that the test was not performed.

## 3 Discussion

Chlorpheniramine maleate,a classic histamine H1 receptor antagonist,In clinical practice,the agent was primarily indicated for antiallergic use and the management of the common cold. Following systemic absorption,it was rapidly assimilated into the circulation, with a high plasma protein binding rate and an extended half-life. Its established therapeutic concentration range was 10–17 ng/mL,and the reported lethal concentration range was 500–1,100 ng/mL (Winek et al., 2001). In mild drug poisoning cases, it mainly presents with central nervous system depression. In moderate to severe poisoning, patients may exhibit symptoms such as hallucinations, hypertension, tachycardia, organ dysfunction, and even respiratory and circulatory depression, which can be fatal (Karamanakos et al., 2004; Guo et al., 2022). Previous reports have indicated that non-traumatic rhabdomyolysis is a rare complication of antihistamine use (Guo et al., 2022; Basha et al., 2025; Kim et al., 2010). The underlying mechanism may involve antihistamines altering skeletal muscle cell membrane permeability, leading to increased intracellular sodium (Na^+^) concentrations and subsequent activation of Na^+^-K^+^-ATPase. This activation results in excessive intracellular adenosine triphosphate (ATP) consumption. Concurrently, elevated intracellular Na+ concentrations induce an increase in intracellular calcium (Ca^2+^) levels, which activates intracellular proteases. Collectively, these mechanisms cause excessive myofibril contraction and energy depletion, ultimately leading to skeletal muscle cell injury and necrosis (Yang et al., 1993; Bosch et al., 2009; Emadian et al., 1996). Rhabdomyolysis refers to a group of clinical syndromes caused by skeletal muscle cell breakdown following injury, characterized by the release of intracellular contents into the systemic circulation; acute kidney injury is a major complication. It can be triggered by various factors, including trauma, infection, electrolyte imbalances, epilepsy, and drug poisoning. Studies have shown that more than 150 types of drug poisoning can induce rhabdomyolysis, including antihistamines (Haas et al., 2003). In this case, the patient presented with persistently elevated CK levels exceeding 10,000 U/L following a single large oral ingestion of chlorpheniramine maleate. Notably, hsTnT and CK-MBmass levels did not increase concurrently, indicating that the injury was localized to skeletal muscle tissue rather than cardiac tissue—findings consistent with the diagnosis of rhabdomyolysis. Additionally, chlorpheniramine was detected in the patient’s blood. Early aggressive management, including gastrointestinal decontamination, diuresis, fluid resuscitation, and maintenance of electrolyte balance, was initiated. This comprehensive treatment yielded favorable outcomes, and the patient was discharged after clinical recovery.

In conclusion, chlorpheniramine poisoning may induce rare rhabdomyolysis. In prior case reports,the majority of chlorpheniramine poisoning cases involved ingested doses ranging from 200 mg to 2,400 mg.A single case report also documented that a patient who had orally ingested 4,000 mg of chlorpheniramine developed rhabdomyolysis (creatine kinase: 195,489 U/L) and acute kidney injury (serum creatinine: 150.1 µmol/L); however, relatively few such case reports were available in the existing literature (Karamanakos et al., 2004; Monte et al., 2010). Currently,the pathogenic mechanism by which antihistamines induce rhabdomyolysis had not yet been fully elucidated, However,in terms of management,the treatment principles for chlorpheniramine poisoning-associated rhabdomyolysis are consistent with those for rhabdomyolysis caused by other etiologies. The primary therapeutic modalities included elimination of the underlying etiology, fluid resuscitation, diuresis,and blood purification. Fluid resuscitation and diuresis not only replenished intravascular volume and increased renal blood perfusion but also elevated the glomerular filtration rate, enhanced myoglobin excretion,and prevented the formation of myoglobin casts within the renal tubules. Urinary alkalinization with bicarbonate mitigated myoglobin-induced renal toxicity and reduced the precipitation of myoglobin casts in the renal tubules, thereby achieving the objective of decreasing the risk of acute kidney injury (Zhou et al., 2025). This case report holds certain reference value for the clinical management and research of chlorpheniramine poisoning. Further exploration and research are warranted for this rare complication of rhabdomyolysis secondary to chlorpheniramine poisoning.

## Data Availability

The raw data supporting the conclusions of this article will be made available by the authors, without undue reservation.
